# Field trial analyses of wheat and cassava benefit from spatial correction

**DOI:** 10.1371/journal.pone.0354968

**Published:** 2026-08-19

**Authors:** Tesfahun Alemu Setotaw, Christine Mwende Nyaga, David James Waring, Jianli Chen, Chiedozie Egesi, Katherine Frels, Lucia Gutierrez, Jinha Jung, Michael Kanaabi, Margaret Krause, R. Esten Mason, Mohsen Mohammadi, Eder Jorge de Oliveira, Eric Olson, Jessica E. Rutkoski, Nicholas Santantonio, Clay Sneller, Mark E. Sorrells, Lukas A. Mueller, Jean-Luc Jannink

**Affiliations:** 1 Plant Breeding and Genetics Section, School of Integrative Plant Science, Cornell University, Ithaca, New York, United States of America; 2 Boyce Thompson Institute, Ithaca, New York, United States of America; 3 University of Idaho, Plant Sciences, Aberdeen, Idaho, United States of America; 4 National Root Crops Research Institute (NRCRI), Cassava Breeding, Umuahia, Nigeria; 5 International Institute of Tropical Agriculture (IITA), Cassava Breeding, Ibadan, Nigeria; 6 University of Nebraska-Lincoln, Agronomy and Horticulture, Lincoln, Nebraska, United States of America; 7 University of Wisconsin - Madison, Plant and Agroecosystem Sciences, Madison, Wisconsin, United States of America; 8 Purdue University, Civil and Construction Engineering, West Lafayette, Indiana, United States of America; 9 National Agricultural Research Organization (NARO), Plant Breeding, Entebbe, Uganda; 10 Department of Crop and Soil Science, Oregon State University, Corvallis, Oregon, United States of America; 11 Department of Soil and Crop Sciences, Colorado State University, Fort Collins, Colorado, United States of America; 12 Department of Agronomy, Purdue University, West Lafayette, Indiana, United States of America; 13 Embrapa Mandioca e Fruticultura, Nugene, Cruz das Almas, Brazil; 14 Michigan State University, Plant, Soil and Microbial Sciences, East Lansing Michigan, United States of America; 15 University of Illinois at Urbana-Champaign, Crop Sciences, Urbana, Illinois, United States of America; 16 Virginia Tech, School of Plant and Environmental Sciences, Blacksburg, Virginia, United States of America; 17 The Ohio State University, Horticulture and Crop Science, Wooster, Ohio, United States of America; 18 USDA-ARS, Robert W. Holley Center for Agriculture and Health, Ithaca, New York, United States of America; Fort Valley State University, UNITED STATES OF AMERICA

## Abstract

Spatial variation is a major source of error in agricultural field experiments affecting genotype performance prediction. Implementing statistical models that account for spatial effects can improve the prediction of genotype performance. This study evaluated the impact of the P-spline spatial correction method on the estimation of genetic parameters and AIC values in two distinct crops, wheat and cassava, using four models: Block, Block + Spatial, Block + Marker, and Block + Marker + Spatial. Analyses were performed on data from 115 and 68 trials obtained from the T3/WheatCAP and Cassavabase databases, respectively. As assessed using Cullis heritability estimates and AIC values, the results demonstrated that correcting for spatial variation improved analyses of grain yield, test weight, plant height, powdery mildew, stripe rust, and bacterial streak disease in wheat. Similar improvements were observed in cassava for dry matter content, dry yield, and plant height. However, no improvement was observed for cassava mosaic disease or bacterial blight. These results were consistent whether or not marker effects were fitted in the models. This study demonstrates that incorporating spatial correction into statistical analyses substantially improves the precision of variety evaluation. By accounting for field heterogeneity, spatial modeling complements experimental design and enhances the accuracy of treatment comparisons. Therefore, integrating robust experimental designs with appropriate spatial analyses is essential for achieving optimal precision and reliability in field trial evaluations.

## Introduction

A significant challenge in field experiments is spatial variability, primarily caused by the heterogeneity of soils and topography in experimental fields, necessitating appropriate experimental designs and spatial correction methods to account for it. Implementing spatial correction with suitable experimental designs enhances prediction accuracy [1]. For instance, one study demonstrated that designs considering spatial variation provided a 138% relative advantage compared to a randomized complete block design in wheat (*Triticum aestivum*) [2]. Similarly, Elias et al. [3] highlighted the advantages of using spatial correction in field assessments of cassava (*Manihot esculenta*).

Genomic selection is now a common practice in both commercial and public-sector breeding programs. Wolfe et al. [4] and Juliana et al. [5] offer comprehensive overviews of genomic selection methods used in plant breeding, specifically focusing on cassava and wheat. The accuracy of genomic predictions is influenced by several factors, such as trait heritability, genetic diversity within the training set, marker density, and the level and distribution of linkage disequilibrium among markers and quantitative trait loci [6]. Additionally, the genetic similarity between training and testing populations affects the accuracy of genomic predictions [7], along with the accuracy of phenotyping in the training set during model development [8]. The impact of trait heritability on genomic prediction accuracy has been explored in multiple crops [9]. Research indicates that higher heritability improves genomic prediction accuracy in maize (*Zea mays*) [10, 11], wheat [12], and in pig (*Sus scrofa*) and cattle (*Bos taurus*) populations [13]. In contrast, low predictability was observed for days to flowering in flax (*Linum usitatissimum*), despite the trait’s high heritability [14]. This discrepancy has been linked to potential inaccuracies in heritability estimation [15].

Higher heritability can be achieved by minimizing noise in field experiments. Traditionally, breeders have used experimental designs such as randomized complete block, incomplete block (both simple and alpha lattice), and p-rep designs to account for spatial variation across field rows and columns. However, these designs often assume uniformity within blocks or incomplete blocks, a condition that can be violated, particularly when evaluating many accessions during the early stages of plant breeding programs [16].

Various spatial correction methods have been proposed to address spatial variability in the experimental fields. Bernal-Vasquez et al. [17] highlighted the importance of incorporating row and column information to correct spatial effects in field experiments. Spatial autoregressive models (AR1 x AR1) and two-dimensional spline smoothing techniques have also been recommended to correct spatial trends in field experiments [16, 18, 19]. Another approach is the moving means as a covariate model (MVNG), introduced by Lado et al. [20]. The application of the variance-covariance matrix structure as a post-data treatment strategy to eliminate spatial trends in agricultural field experiments has been reported by [3, 21–23].

The application of spatial correction to specific experiments in plant and animal breeding has been demonstrated by different authors. Spatial correction methods that employed spatial autocorrelation structures have been shown to improve heritability estimates and reduce prediction bias, as demonstrated in lodgepole pine trees (*Pinus contorta*) [24] when compared to estimates obtained without spatial correction. The spatial correction reduced experimental errors, increased genetic parameter values (such as heritability), and reduced prediction bias [16, 19]. In common bean (*Phaseolus vulgaris*) breeding, spatial correction models have been reported to increase heritability, prediction accuracy, and lower prediction error variance [25]. Finally, the application of appropriate spatial correction methods has been shown to enhance genomic prediction accuracy across various crops [17, 20, 26–28]. Just as for the estimates of any genetic parameter, larger sample sizes can improve estimates of spatial effects [1]. We are not aware of research assessing the importance of trial size on the value of spatial analysis. On the one hand, smaller trials might make the analysis less relevant due to the higher probability of trial uniformity. On the other hand, as a trial gets larger, heterogeneity in spatial correlations may arise that would be challenging to model.

Most studies on spatial corrections analyze relatively few trials and do not sample a range of breeding programs and stages, such that they may not provide robust assessments of the analysis impact. Evaluating a model using many datasets increases the accuracy of the genetic parameter estimates [29], enables broader generalization, helps uncover potential weaknesses, and broadens its applicability across different testing scenarios [30]. Databases like The Triticeae Toolbox (T3) for the Wheat Coordinated Agricultural Project (WheatCAP, https://triticeaecap.ucdavis.edu) and Cassavabase (the database of the Next Generation Cassava Breeding project, https://www.nextgencassava.org) were developed to store molecular and phenotypic data collected by breeders using the Breedbase platform [31]. These resources offer researchers valuable opportunities to test and validate new data analysis methods over many trials using diverse datasets collected over multiple years and locations by several breeding programs.

This study compares four analytical models (Block, Block + Spatial, Block + Marker, Block + Marker + Spatial) in terms of heritability and model fit for agronomic and disease traits using trial data from two Breedbase instances [31]: the Wheat Coordinated Agricultural Project (T3/WheatCAP, https://wheatcap.triticeaetoolbox.org and https://wheat.triticeaetoolbox.org) and the Next Generation Cassava Project (Cassavabase, https://www.cassavabase.org).

## Materials and methods

### Phenotype data

The wheat datasets were obtained from the WheatCAP project database (https://wheatcap.triticeaetoolbox.org). A total of 115 trials were used, of which 18 had genome-wide marker information. Trials selected had row and column field layout information. The 18 trials with marker information were genotyped with the Illumina Infinium 90k genotyping platform, which generated 34,135 markers. Three agronomic traits (grain yield, plant height, and grain test weight) and seven disease traits [bacterial streak (*Xanthomonas translucens* pv. *undulosa*), stripe rust (*Puccinia striiformis* f. sp. *tritici*) response, stripe rust severity, barley yellow dwarf virus (BYDV, family *Luteoviridae*) response, BYDV severity, *Fusarium* head blight (*Fusarium* spp.), and powdery mildew (*Blumeria graminis* f. sp. *tritici*)] were considered.

The cassava datasets were obtained from Cassavabase (https://cassavabase.org). Three agronomic traits (dry yield, plant height, and dry matter content) and two disease traits (cassava bacterial blight (*X. axonopodis* pv. *manihotis*) and cassava mosaic disease (*Begomovirus* spp.)) were evaluated. For the agronomic and disease traits, a total of 57 and 11 trials were used, respectively. Each trial had more than 30 unique accessions with row and column field layout information. Marker information (on average, 17,500 markers) was available for 33 trials out of the 57 trials for agronomic traits and 3 out of the 11 trials for disease traits. For both wheat and cassava, the agronomic and disease traits were selected based on the availability of the measurements in the selected trials. All the agronomic and disease data were collected according to the methods described in the trait ontologies (https://cropontology.org/) [32].

The list of trials, number of accessions, number of plots per trial, and average number of replications for wheat and cassava are presented in S1 Table. The cassava data is fully available at https://cassavabase.org/. Most of the wheat data is available at https://wheat.triticeaetoolbox.org/. Some of the data, however, is still embargoed at https://wheatcap.triticeaetoolbox.org/. That data will become available within the next year.

### Data analysis

#### Phenotypic data cleaning.

The raw data of measured traits underwent descriptive statistical analysis and outlier removal. Outliers were identified and eliminated using r-studentized t-test statistics [33]. The r-studentized values were calculated with the rstudent function in R software (R Core Team 2023). Observations with absolute studentized residual values greater than 3 were deemed outliers and excluded from further analysis. We also estimated the heritability of the traits prior to removing outliers to assess whether their removal influenced the estimated parameters.

Before subsequent analyses, each trial was partitioned into six incomplete blocks: three aligned with the longer dimension (rows/columns) and two with the shorter dimension. We set up this post-hoc incomplete block structure because:

1. We did not always trust the experimental design designation given by breeders in the trial metadata. It is often challenging to collect the correct design when uploading trials.
2. We did not want to analyze the trials as completely randomized or randomized complete block trials without accounting for any field heterogeneity. Such an analysis would have made a conclusion of the benefit of spatial analysis too obvious.
3. This approach allowed us to analyze all of the trials together using unified statistical models that simplified downstream interpretation. These unified statistical models were then fitted to each trial (see Data analysis below), rather than applying separate design-specific models to individual trials.

#### Genomic data acquisition and data cleaning.

Genotyping data were obtained as VCF files from the WheatCAP and Cassavabase databases according to their respective genotyping protocols. Most of the trials considered were genotyped using the 90k Illumina SNP chip (wheat) and/or genotyping-by-sequencing (GBS) (wheat and cassava) protocols [34]. The SNP markers were filtered using a minor allele frequency (MAF) of 0.05, a maximum missing value within individuals of 20%, and a maximum heterozygosity value of 20% and 70% for wheat and cassava [35], respectively, using TASSEL software [36]. The filtered data was imputed for the missing values using the LDKNNI imputation method [37] using TASSEL.

#### Data analysis.

To estimate the additive relationship matrix, the imputed VCF file was converted into a dosage matrix with vcftools [38]. The additive relationship matrix for the genotypes in each trial, K, was calculated using the A.mat function from the Sommer package [39]. To prevent the singularity issue in the matrix, K was adjusted as K = (1-0.05) x K + 0.05 x diag(K) [12]. In trials where accessions were genotyped with two or more protocols, a distinct relationship matrix was computed for each protocol, and these matrices were subsequently merged using the CovComb function from the CovCombR package for R [40].

#### Models compared.

We considered four data analysis models: Block, Block+Spatial, Block+Marker, and Block+Marker+Spatial (Table 1).

**Table 1:** The four analysis models or scenarios implemented in the study.

	With marker data a ~ N(0, Kσ_a_^2^)	Without marker data u ~ N(0, Iσ_u_^2^)
With spatial	Y = **X**β + **B**b **+ Z**a + **S**s + **I**e	Y = **X**β + **B**b + **Z**u + **S**s + **I**e
Without spatial	Y = **X**β + **B**b + **Z**a + **I**e	Y = **X**β + **B**b + **Z**u + **I**e

Where X is the design matrix of the fixed effects (here, only the grand mean, β), Z is the design matrix of the random effect of accessions given by a or u depending on the presence/absence of marker data (Table 1), and e ~ N(0, σ_e_^2^I) where I is the identity matrix and K is the additive genomic relationship matrix. Finally, the spatial components (Ss) include S, the B-spline design matrix, and s, the random spline coefficient, with s ~ N(0, σ_s_^2^P) where P is the generalized inverse of the penalty matrix [19]. B is the design matrix of the random effect block, and b is the random block effect b ~ N(0,σ_b_^2^I).

#### Parameters for model comparison.

To compare the four models, heritability and AIC (Akaike information criteria) values were estimated for each trial. To evaluate the impact of outliers on parameter estimates, analyses were conducted both with and without outlier removal.

#### Heritability.

To estimate the broad- and narrow-sense heritability of the traits of interest, the variance components were estimated by fitting the models indicated in Table 1 using the mmer function of the sommer package for R [39]. The spatial analysis used the mmer helper function spl2Da, “Two-dimensional penalised tensor-product of marginal B-Spline basis”.

The heritability of the traits was estimated according to [41], called the Cullis heritability. The Cullis heritability is estimated as follows:

$H^{\text{2}}=\text{1}\;-\frac{A_{tt}}{\left({\text{2}\sigma }_{u}^{\text{2}}\right)}$, where $H^{\text{2}}$ is the Cullis heritability, $\sigma_{u}^{\text{2}}$ is the genetic variance, and $A_{tt}=\frac{\text{2}}{t-\text{1}}(tr(C^{tt})\;-\frac{\text{1}}{t}{\text{1}}_{t}^{\prime }C^{tt}{\text{1}}_{t})\;$ is the average pairwise prediction error variance of the accessions derived from $C^{tt}$, which is the scaled prediction error variance matrix of the accession effects, all estimated without using marker information. $C^{tt}$ is obtained as an output of the Sommer package. The narrow-sense heritability was estimated as

$h^{\text{2}}=\text{1}\;-\;\frac{A_{aa}}{\left({\text{2}\sigma }_{a}^{\text{2}}\right)}$, where $h^{\text{2}}$ is the plot-level narrow-sense heritability, $\sigma_{a}^{\text{2}}$ is the additive genetic variance estimated based on the marker information, where $A_{aa}\;$ is calculated as $A_{tt}$, but with $C^{tt}$ obtained from an analysis using marker information.

To compare the impact of spatial correction on heritability estimates, the heritability difference was estimated between Block vs. Block+Spatial and Block+Marker vs. Block+Marker+Spatial.

The strength of the spatial effect for each trial, γ, was estimated using the formula:


$$\begin{array}{c} \gamma =\frac{\sigma_{s}^{\text{2}}\;}{\sigma_{e}^{\text{2}}\;} \end{array}$$

where $\sigma_{s}^{\text{2}}\;$ is the spatial variance, and $\sigma_{e}^{\text{2}}$ error variance without spatial correction.

#### AIC (Akaike Information Criterion).

The AIC value was estimated as follows:

$AIC=-\text{2}ln\left(\hat{L}\right)+\text{2}P$ where P is the number of estimated parameters in the model, and $\hat{L}$ is the log-likelihood for the model.

The impact of spatial correction on model fitness was assessed by calculating the AIC value difference between the models Block vs. Block+Spatial and Block+Marker vs. Block+Marker+Spatial.

## Results

### Heritability and variance components

**Fig 1:**
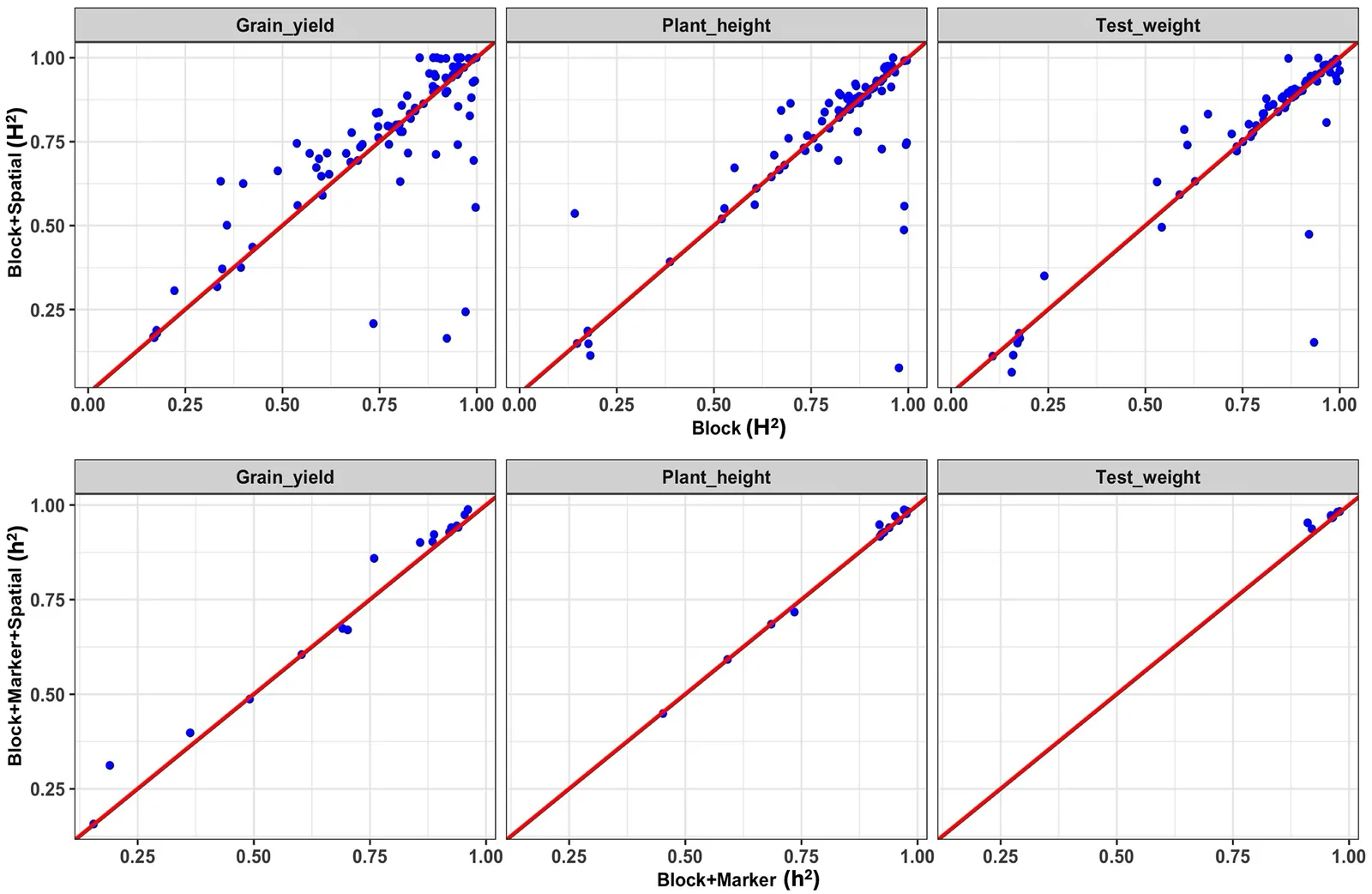
Wheat agronomic traits: Scatterplots comparing the broad-sense heritability (H^2^) using the Block vs Block+Spatial models (A) and the narrow-sense heritability (h^2^) using the Block+Marker vs Block+Marker+Spatial models (B).

**Fig 2:**
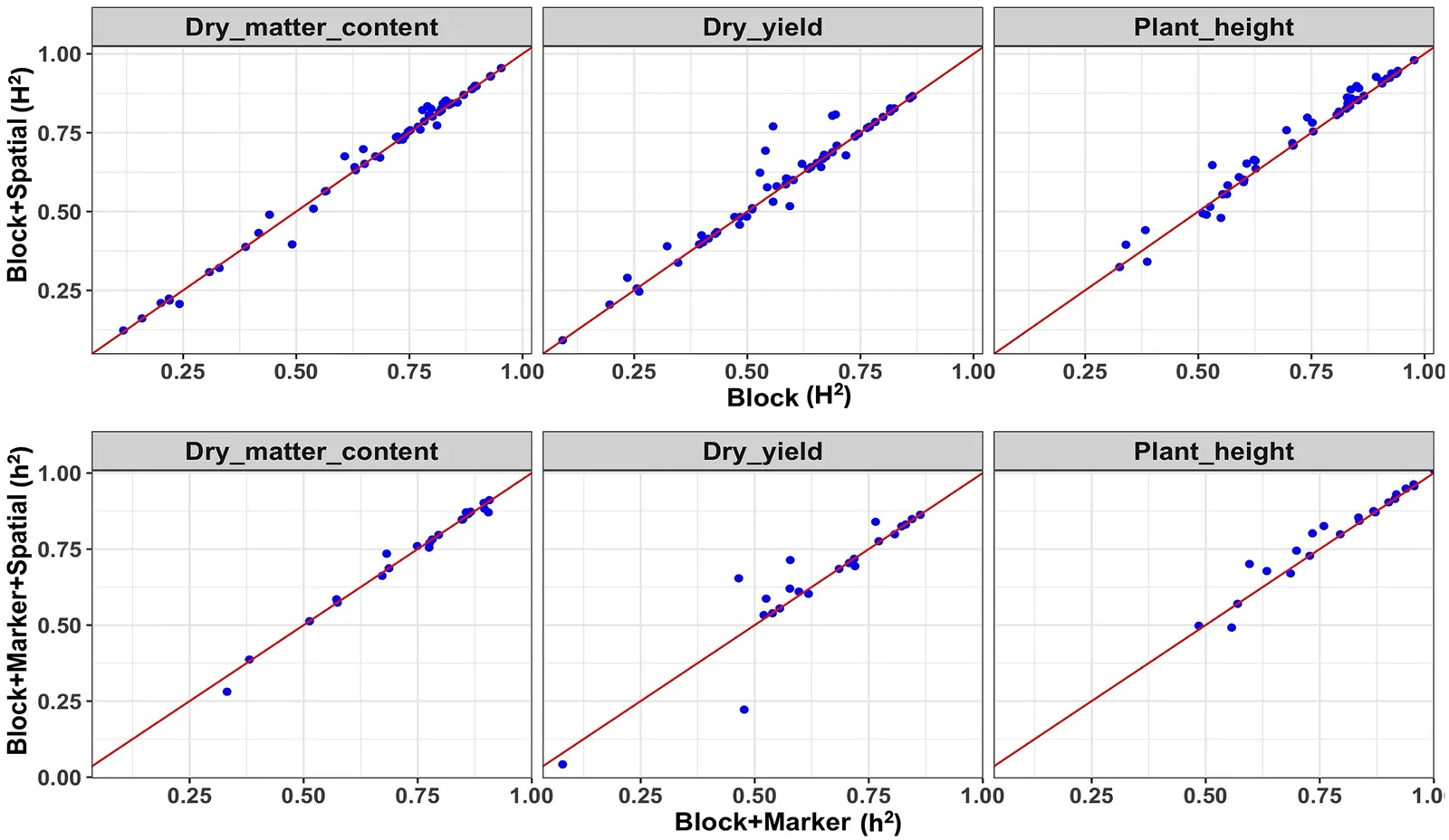
Cassava agronomic traits: Scatterplots comparing the heritability (H^2^) using Block vs Block+Spatial (A) and Block+Marker vs Block+Marker+Spatial (B) in cassava trials.

Heritability estimates ranged from 0.0 to 1.0 for both wheat and cassava. Across most trials, spatial correction applied with or without marker information improved heritability estimates for agronomic traits in both crops (Fig 1 and Fig 2). The greatest differences in heritability were observed in wheat. Specifically, 70% of wheat trials showed increased heritability following spatial correction across traits (Fig 1). The largest improvements in broad-sense heritability for wheat were recorded for plant height (H² = 0.35) and grain yield (H² = 0.29) under the Block + Spatial model (Fig 1). In cassava, the highest gains in broad-sense heritability were observed for dry yield and plant height (Fig 2). However, spatial correction negatively impacted heritability in some trials for both crops (Fig 1 and Fig 2), with the greatest reductions occurring for grain yield in wheat and plant height in cassava. The extremely low heritability observed for some trials under spatial correction compared to models without spatial adjustment may be caused by spatial adjustment capturing some of the genetic variance and increasing the prediction error variance. We believe that this occurs when the randomization causes some confounding between spatial and genetic effects: that is, when accessions with similar genetic effects are by chance randomized to be planted close to each other [42].

**Fig 3:**
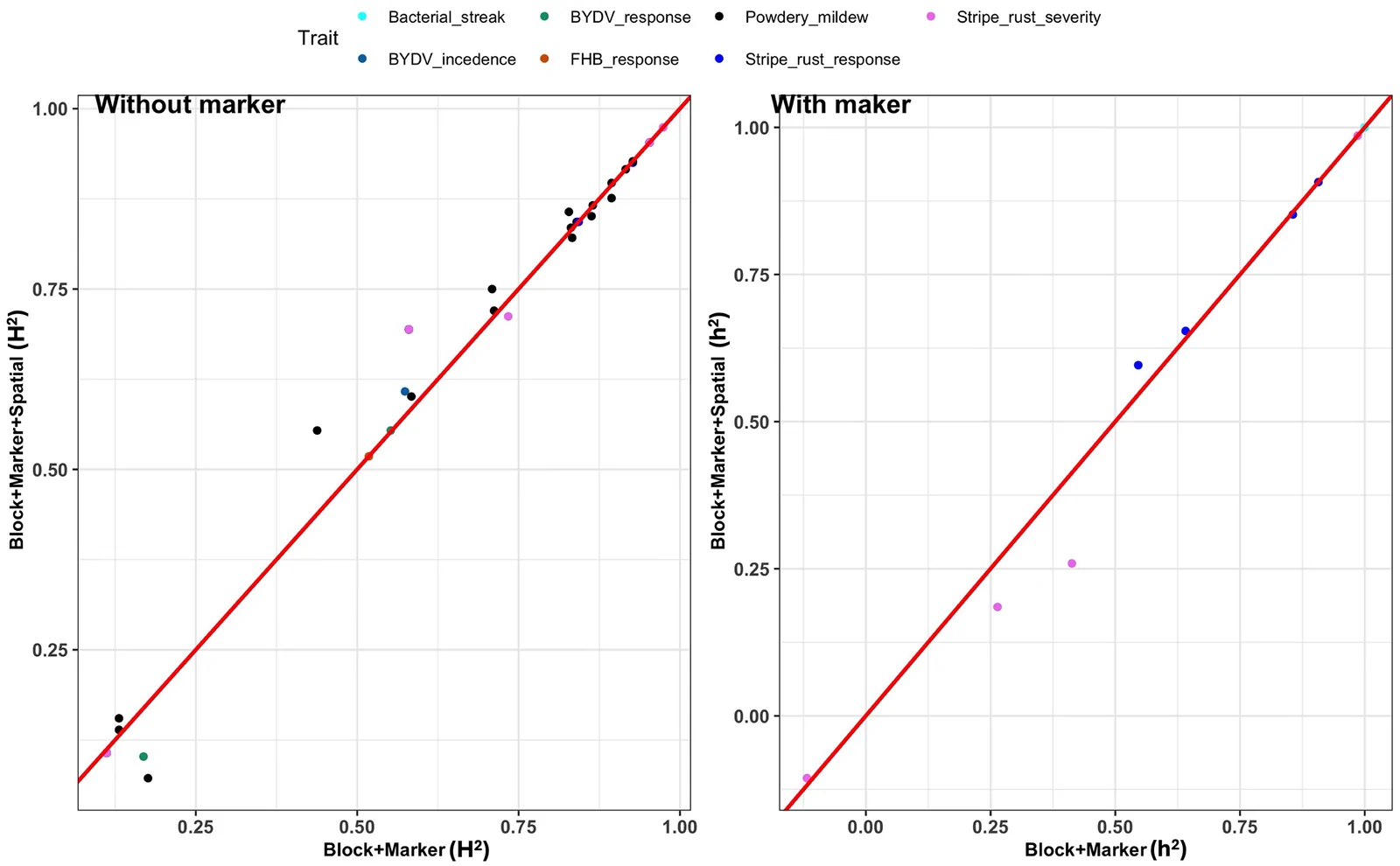
Wheat disease traits: Scatterplots comparing the broad-sense (H^2^) and narrow-sense heritability (h^2^) using Block vs Block+Spatial and Block+Marker vs Block+Marker+Spatial. BYDV and FHB refer to Barley Yellow Dwarf Virus and Fusarium Head Blight, respectively.

**Fig 4:**
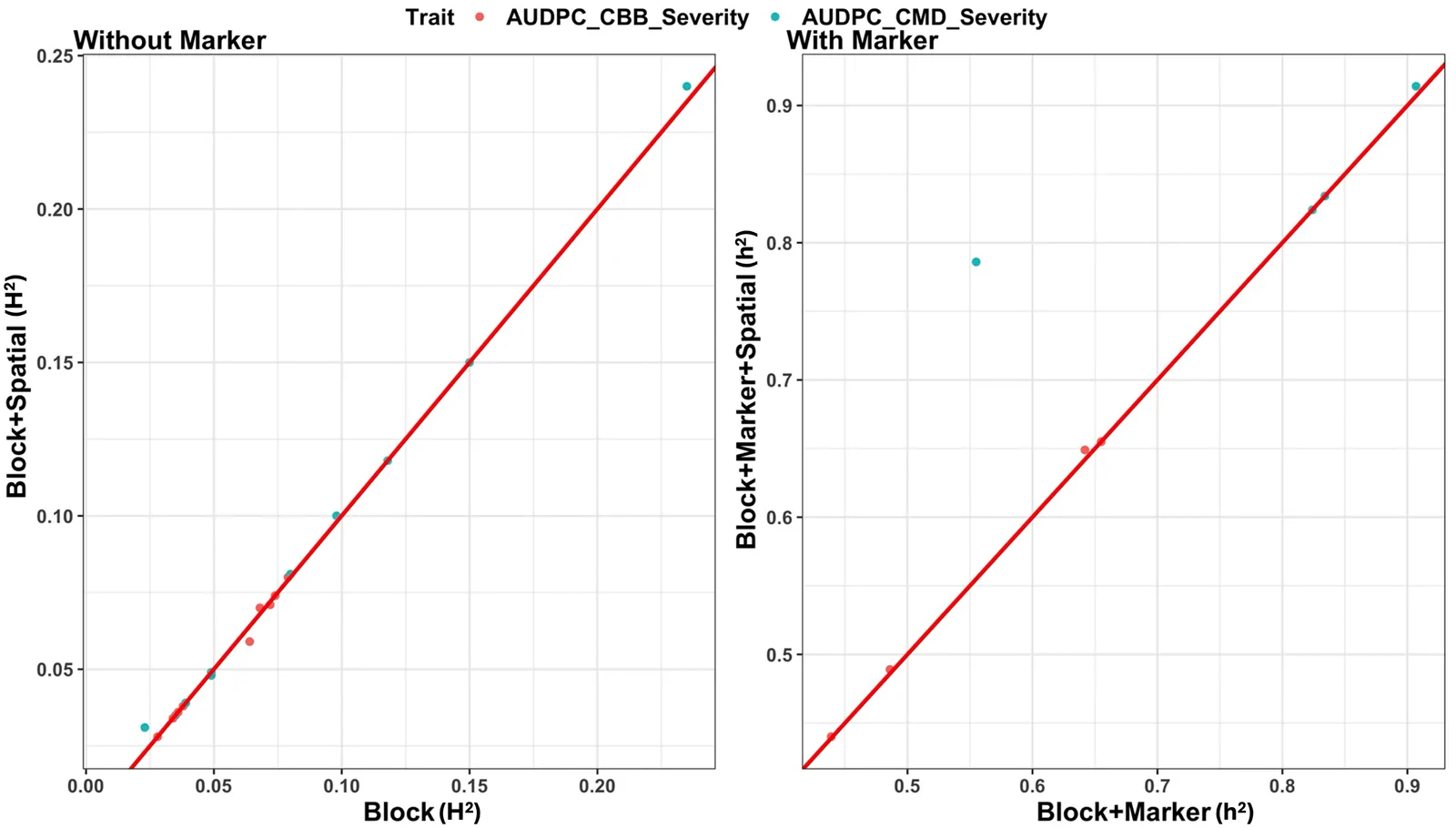
Cassava disease traits: Scatterplots comparing the heritability (H^2^) using Block vs Block+Spatial (A) and Block+Marker vs Block+Marker+Spatial (B). AUDPC is the Area Under the Disease Progress Curve.

The average improvement in narrow-sense heritability ranged from 0% to 13% and 0% to 7% under the spatial correction for wheat and cassava, respectively. In wheat, the maximum narrow-sense heritability improvement was observed for grain yield (13%), test weight (4%), and plant height (3%) (Fig 1), whereas in cassava, the maximum improvement was obtained for dry yield and dry matter content (Fig 2). In wheat, the narrow-sense heritability improvement under spatial correction was observed in the majority of the trials. Among the disease traits, the maximum increase in heritability estimate was recorded for bacterial streak, followed by powdery mildew (Fig 3) in wheat. While in cassava, no improvement in heritability was observed for evaluated disease traits (Fig 4). In wheat, the maximum narrow-sense heritability improvement was observed for stripe rust and bacterial streak (Fig 3). The highest spatial strength (γ) was obtained for grain yield, followed by test weight for the model with marker data in wheat (S1 Fig). The increase in heritability was associated with the increase in the spatial effect strength (γ) for all traits evaluated in wheat (S1 Fig) and cassava with or without marker information. To assess whether outlier removal influenced our results and conclusions, we estimated heritability and AIC values without excluding outliers. No significant differences were observed in narrow- or broad-sense heritability, regardless of whether spatial correction was applied in wheat and cassava (S2–S5 Figs). We also assessed the relationship between the number of plots in an experiment and the estimated parameters, and we found that there was no clear relationship between the number of plots per trial and heritability estimates for wheat and cassava.

### Model comparison

**Fig 5:**
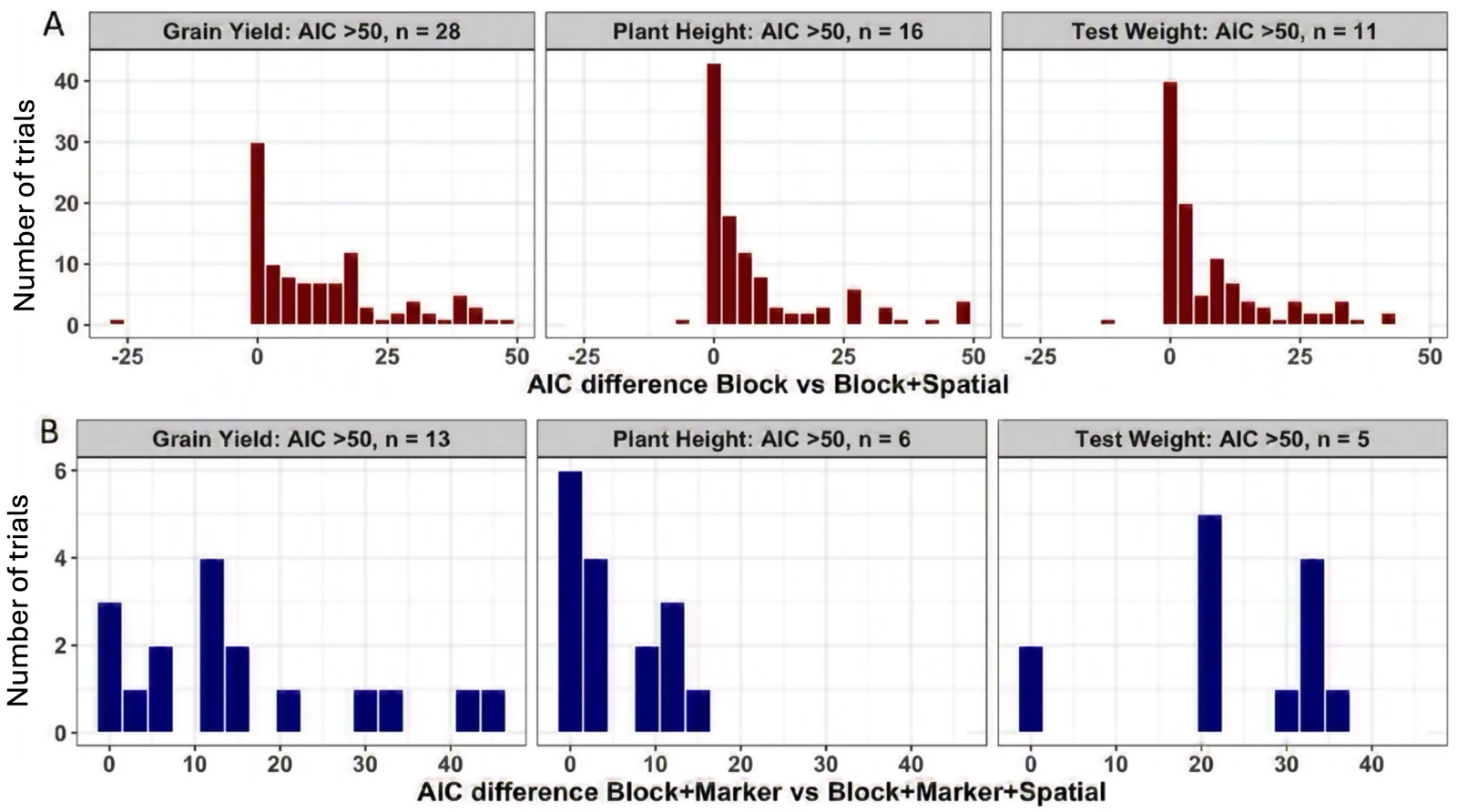
The differences in AIC values for Block vs. Block+Spatial (A) and Block+Marker vs. Block+Marker+Spatial (B) models for agronomic traits in wheat. Trials where the difference in AIC values was greater than 50 were not plotted. The number of such trials is given in the plot header.

**Fig 6:**
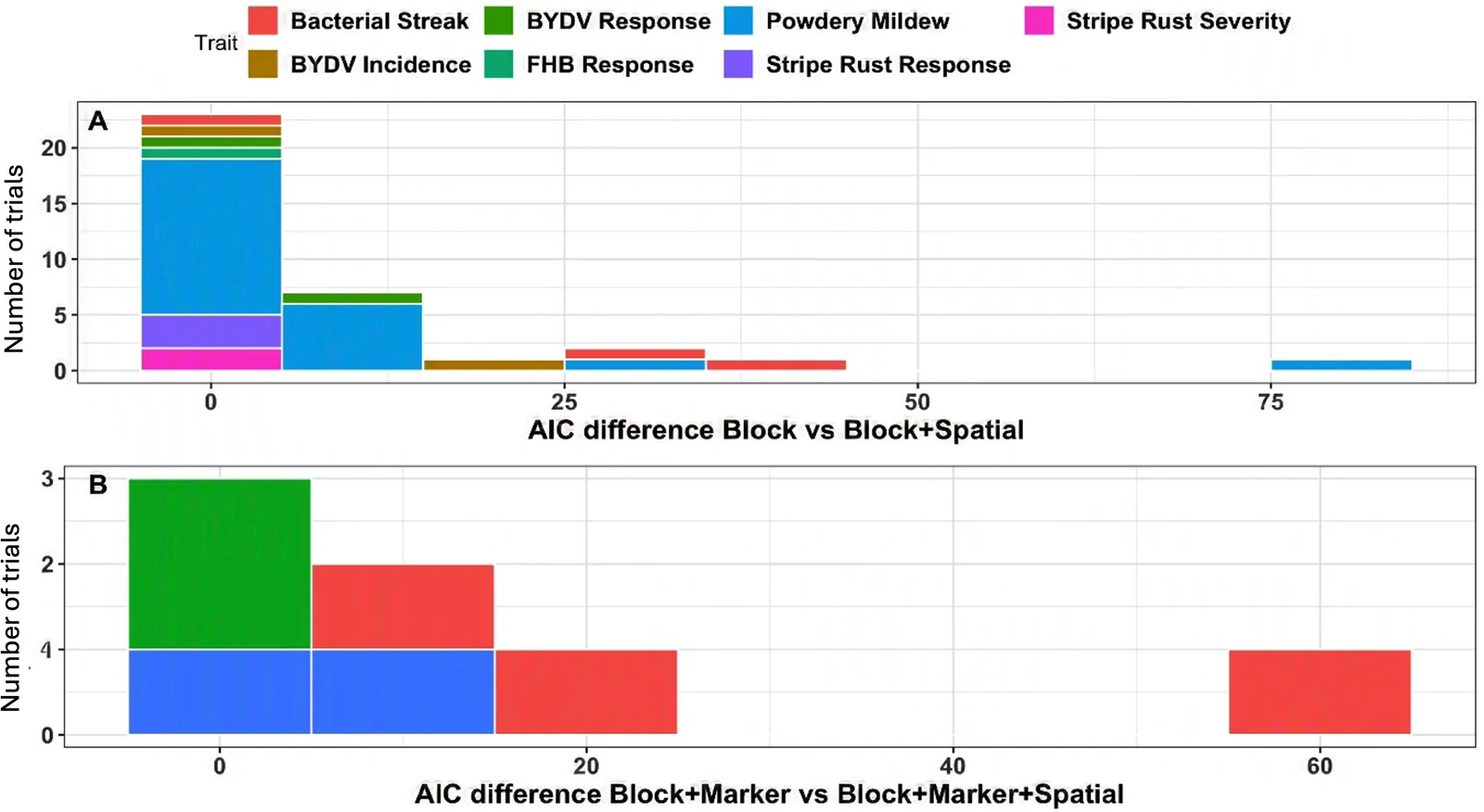
The differences in AIC values for Block vs. Block+Spatial (A) and Block+Marker vs. Block+Marker+Spatial (B) models for disease traits in wheat. BYDV and FHB stand for Barley Yellow Dwarf Virus and Fusarium Head Blight, respectively.

**Fig 7:**
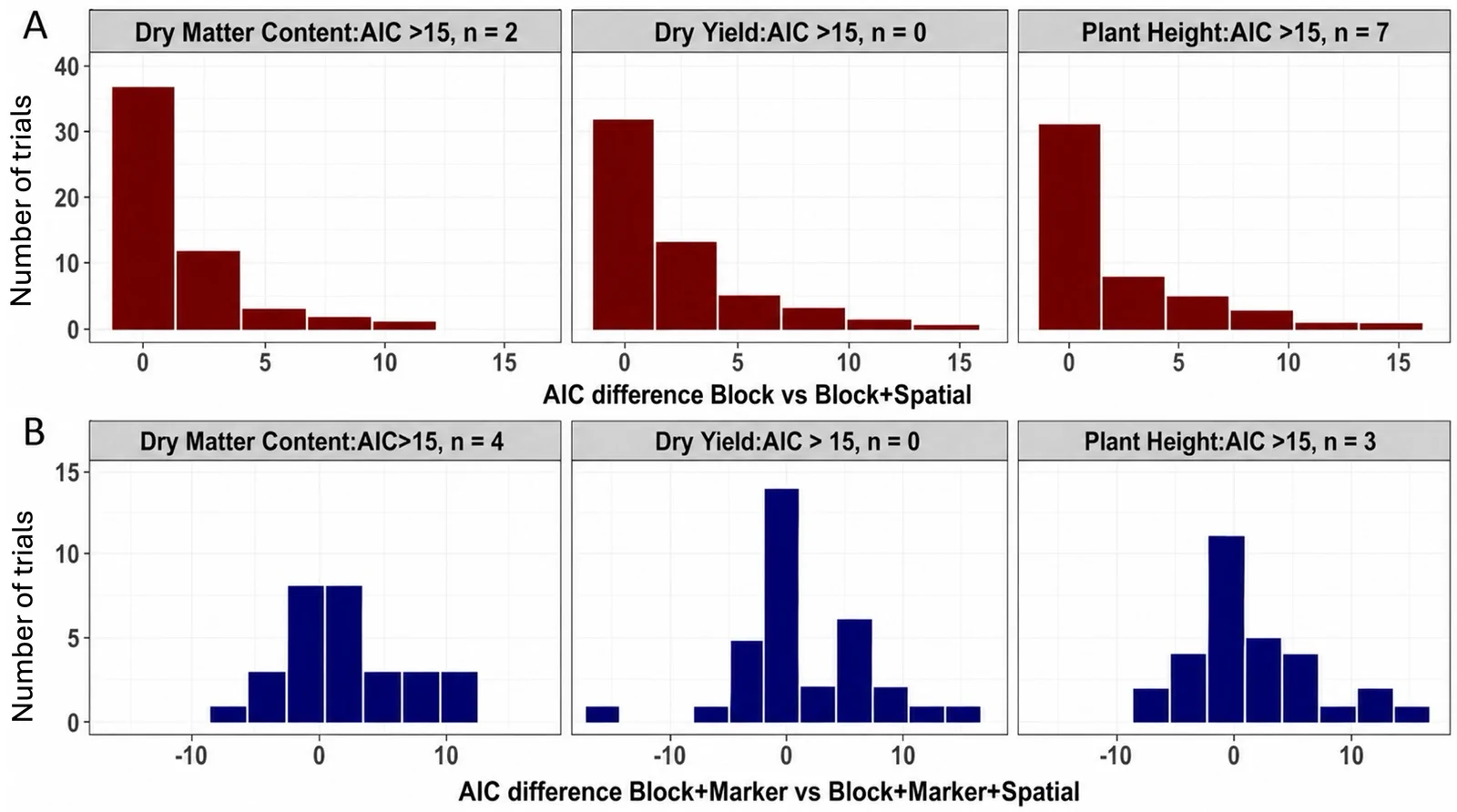
The differences in AIC values of Block vs. Block + Spatial (A) and Block + Marker vs. Block +Marker + Spatial (B) models for agronomic traits in cassava. Trials where the difference in AIC values was greater than 15 were not plotted. The number of such trials is given in the plot header.

**Fig 8:**
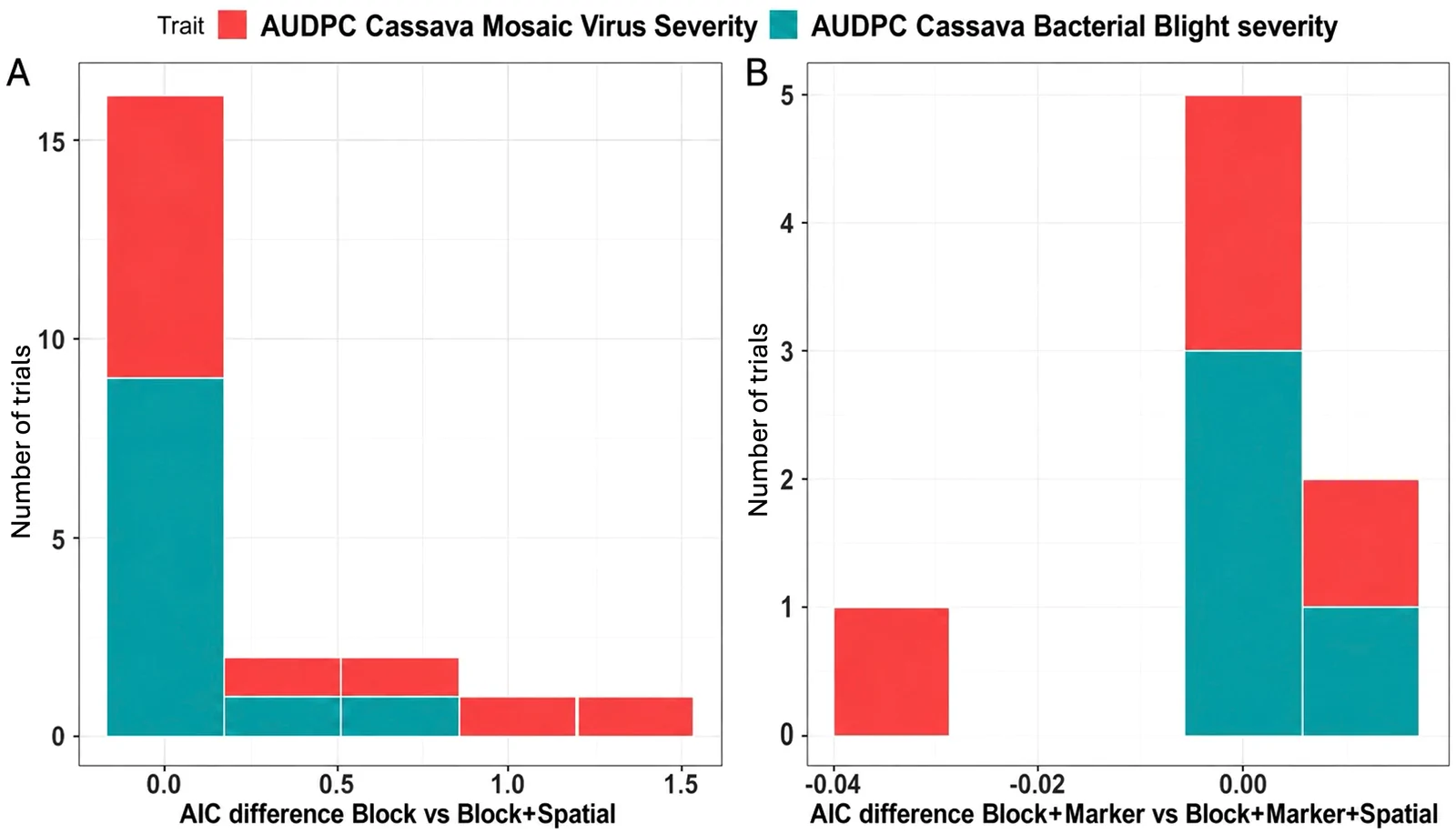
The differences in AIC values of Block vs. Block + Spatial (A) and Block + Marker vs. Block +Marker + Spatial (B) models for disease traits in cassava. AUDPC is Area Under the Disease Progress Curve.

We compared four models, including Block, Block + Spatial, Block + Marker, and Block + Marker + Spatial. Models with spatial correction had lower AIC values than models without spatial correction in the majority of the trials for cassava and wheat (Figs 5–8). The Block + Marker + Spatial model performed well for cassava and wheat for the agronomic traits at all the locations. The largest AIC value difference was observed for the Block+Marker+Spatial model compared to the other three models in cassava and wheat.

For agronomic traits, cassava had a greater number of trials with differences in AIC values below zero compared to wheat (Fig 5 and 7). In cassava, 11 and 4 trials out of 57 trials showed negative differences in AIC values for the Block vs. Block+Spatial and Block+Marker vs Block+Marker+Spatial models (Fig 7), respectively. Whereas in wheat (Fig 5), 9 trials out of 115 showed negative differences in AIC values for Block vs Block+Spatial.

For disease traits in wheat, the maximum difference in AIC values was observed for bacterial streak for Block vs. Block+Spatial and Block+Marker vs. Block+Marker+Spatial (Fig 6), whereas in cassava, the difference in AIC values for all the disease traits did not exceed 2 (Fig 8). In wheat, the maximum difference in AIC value was observed for Block vs. Block+Spatial (Fig 6) compared with Block+Marker vs. Block+Marker+Spatial. In general, more trials with high difference in AIC values were observed in wheat than in cassava.

The evaluated models in wheat showed reductions in AIC values for models with spatial corrections as compared to trials without spatial corrections for all traits except stripe rust response. When considering average heritability estimates, the Block+Marker+Spatial model was the most suitable for grain yield, plant height, and stripe rust response, while the Block+Spatial model performed best for test weight, bacterial streak, and stripe rust severity. It is important to note that the heritability estimates for the Block+Marker and Block+Marker+Spatial models represent narrow-sense heritability, which is inherently less than or equal to broad-sense heritability. In general, for both wheat and cassava, the Block + Marker+Spatial and Block+Spatial scenarios outperformed the scenarios without spatial correction.

The effect of the number of plots per trial on model AIC values did not display a consistent pattern in wheat. In cassava, however, AIC values increased with the number of plots per trial, which may be related to the larger plot size and the growth characteristics of cassava as a root crop.

## Discussion

### Improvement in heritability due to spatial correction

We adopted the P-spline smoothing approach for spatial adjustment, as proposed by [43] and [19], as an alternative to the widely used AR1 × AR1 model. Previous reports have highlighted convergence issues when fitting AR1 × AR1 spatial models [23]. In contrast, the P-spline framework offers a key advantage: most variance–covariance structures are linear in their parameters, which facilitates model fitting and reduces convergence problems. Moreover, the P-spline method enables simultaneous modeling of global and local spatial trends through a smooth surface representation, providing greater flexibility in capturing spatial heterogeneity [44].

This study demonstrated the improvement in heritability estimates for some of the traits across different trials under the spatial correction for wheat and cassava. This finding aligns with those reported by [44], where spatial correction improved heritability estimates in sorghum (*Sorghum bicolor*). The increase in heritability estimates was associated with a reduction in experimental error, enabling more accurate prediction of genotype performance [24]. In Lado et al. [20], the use of moving means as a covariate for spatial correction in wheat trials provided an increase in heritability for grain yield and thousand seed weight compared to an incomplete block design with row-column information, which is consistent with our findings (Fig 1). Similarly, heritability improvement was reported with spatial correction in common bean breeding [25]. The findings of this study align with those of various crops, the authors of which advocate for incorporating spatial correction in data analysis to enhance heritability and minimize prediction bias [26, 28, 45]. However, contrary to our results, [26] reported no improvement in heritability estimates for cassava with spatial correction, possibly because they used a different spatial correction method.

The difference in how much spatial correction improved heritability estimates for disease traits in wheat and cassava can be attributed to differences in the nature of disease dispersal mechanisms between the two species. In wheat, rust disease-causing agents or spores are mostly dispersed through wind, rainfall, crop residue, mechanical damage, and root-feeding insects [46], [47, 48]. Therefore, most diseases in wheat are affected by the field conditions [46], which renders spatial correction important for accurate parameter estimation. Some mechanisms of disease transmission in cassava are similar, for example, wind, rain, and insect vectors. Specifically, whiteflies [49] transmit cassava mosaic disease, while wind and rain or contaminated farm tools [50, 51] transmit cassava bacterial blight. However, most cassava diseases are also transmitted by vegetative propagules. This mode of transmission does not depend on the soil conditions of the field experiment [50, 51]. Thus, in cases where disease incidence originates from infected vegetative propagules used for trial establishment, the resulting variation reflects the initial distribution of inoculum rather than spatially structured environmental heterogeneity. In cassava, major viral diseases such as Cassava Mosaic Disease and Cassava Brown Streak Disease are well known to be transmitted through infected stem cuttings used for planting [52]. Consequently, spatial correction methods, which are designed to model field-based environmental gradients, are unlikely to be effective and will not substantially improve heritability estimates for disease traits under such conditions. If possible, it may be valuable for cassava breeders to record disease values of the plots from which vegetative propagules originate and to use those values as covariates in new trials. Our study revealed a lower spatial-to-error variance ratio for diseases when compared to agronomic traits in cassava and wheat, indicating that agronomic traits are more affected by soil spatial heterogeneity (Fig 2 and S1 Fig).

### Model comparison

We compared four models to evaluate their effects on heritability and the Akaike Information Criterion (AIC). Heritability determines the portion of phenotypic variation attributed to genetic factors, whereas AIC assesses model complexity, favoring models that achieve both accuracy and simplicity. The Block+Marker+Spatial and Block+Spatial models yielded lower AIC values than models that lacked the spatial component across most trials for all agronomic traits in cassava and wheat. For these traits, including the spatial effect eliminated more variation from the error variance, offsetting the penalty typically incurred for adding extra parameters to the model. Consistent with our findings, previous reports have noted improvements in model selection criteria when implementing spatial correction in sorghum [16, 44]. The addition of spatial correction generally reduced error variance components, resulting in higher heritability estimates and lower AIC values. This reduction correlated with an increase in heritability compared to the models lacking spatial information. While AIC serves as a robust measure of overall model improvement, heritability reflects the proportion of phenotypic variance attributed to genetic influences. If the spatial correction removes proportionally more variance from the error than the genetic term, it will increase the estimated heritability even if the AIC value suggests that the spatially corrected model is inferior. Nevertheless, our results demonstrate that both AIC and heritability typically benefit from seeking to capture variance due to spatial effects.

When considering cassava, the relevance of spatial correction in estimating genetic parameters and reducing prediction error has been documented previously [26], thereby supporting our findings. The improvement of genetic parameter estimates due to spatial correction has also been noted in studies involving lodgepole pine trees [24] and maize [27]. Bernal-Vasquez et al. [17] found that simpler models that integrated row and column information led to higher genomic prediction accuracies than more complex spatial analysis models like AR1 × AR1 and splines. We did not test such simple models, but the enhancement of genetic parameter estimates and reduction in AIC values with spatial correction for agronomic traits in wheat and cassava demonstrated the benefit of spatial adjustments for accurate prediction of quantitative traits influenced by macro- and micro-environmental field conditions.

### Study limitations

This study has several limitations that should be acknowledged. First, the datasets used were obtained from public databases, and in many cases, detailed metadata describing the original experimental designs (e.g., blocking structure, plot layout, randomization procedures) were incomplete or unavailable. This limitation restricted our ability to explicitly account for and compare the effects of different experimental designs within the spatial correction framework, which may influence the accuracy and interpretation of the results.

Second, although multiple analytical approaches were evaluated, the genomic prediction component of this study relied primarily on the standard GBLUP model. While GBLUP is widely used and provides a robust baseline, it may not capture more complex genetic architectures or non-additive effects that could be better modeled by alternative methods. As a result, the interaction between spatial correction and more advanced genomic models was not explored.

Finally, because the analysis was conducted using heterogeneous datasets collected across different environments, years, and management conditions, there may be unaccounted sources of variability that influence spatial patterns. Although spatial correction methods such as P-splines help mitigate these effects, they may not fully capture all underlying sources of field heterogeneity. Despite these limitations, the scale of the study should enable it to provide robust estimates of the effects of spatial heterogeneity and valuable insights into its role and importance.

## Conclusion

Spatial correction models can estimate field heterogeneity with sufficient accuracy to improve genetic parameter estimates and reduce performance prediction error. For datasets with marker information, the model comparison using AIC showed that the Block + Marker + Spatial model was best in the majority of the trials for agronomic traits in two highly dissimilar crops. Moreover, datasets without marker information also showed the Block + Spatial model to be superior to the Block model for agronomic traits. Finally, vegetative propagation of cassava appeared to have reduced the value of spatial correction for disease traits.

## Supporting information

S1 Fig The relative increase of the heritability estimate in relation to the spatial effect strength with and without marker information. BYDV and FHB refer to Barley Yellow Dwarf Virus and Fusarium Head Blight, respectively.
(TIFF)

S2 Fig Wheat agronomic traits: Scatterplots comparing the broad-sense heritability (H^2^) using the Block vs Block+Spatial models (A) and the narrow-sense heritability (h^2^) using the Block+Marker vs Block+Marker+Spatial models (B) without removing the outlier observations. (TIFF)

S3 Fig Wheat disease traits: Scatterplots comparing the broad-sense (H^2^) and narrow-sense heritability (h^2^) using Block vs Block+Spatial and Block+Marker vs Block+Marker+Spatial. BYDV and FHB refer to Barley Yellow Dwarf Virus and Fusarium Head Blight, respectively without removing the outlier observations.
(TIFF)

S4 Fig Cassava agronomic traits: Scatterplots comparing the heritability (H^2^) using Block vs Block+Spatial (A) and Block+Marker vs Block+Marker+Spatial (B) in cassava trials without removing the outlier observations. (TIFF)

S5 Fig Cassava disease traits: Scatterplots comparing the heritability (H^2^) using Block vs Block+Spatial (A) and Block+Marker vs Block+Marker+Spatial (B). AUDPC is the Area Under the Disease Progress Curve without removing the outlier observations.
(TIFF)

S1 Table List of trials, number of accessions, number of plots per trial, and average number of replications for wheat and cassava. (XLSX)
